# Comparison of the clinical characteristics and mortality of adults infected with human coronaviruses 229E and OC43

**DOI:** 10.1038/s41598-021-83987-3

**Published:** 2021-02-24

**Authors:** Won-Il Choi, In Byung Kim, Sang Joon Park, Eun-Hye Ha, Choong Won Lee

**Affiliations:** 1 Department of Internal Medicine, Myongji Hospital, Hanyang University College of Medicine, 55 Hwasu-ro, 14 beon-gil, Deogyang-gu, Goyang-si, Gyeongji-do 10475 Republic of Korea; 2 Department of Emergency Medicine, Myongji Hospital, Hanyang University College of Medicine, Goyang, Republic of Korea; 3 Department of Occupational and Environmental Medicine, Sungso Hospital, Andong, Republic of Korea

**Keywords:** Microbiology, Medical research

## Abstract

The purpose of the study was to compare clinical characteristics and mortality among adults infected with human coronaviruses (HCoV) 229E and OC43. We conducted a retrospective cohort study of adults (≥ 18 years) admitted to the ward of a university teaching hospital for suspected viral infection from October 2012 to December 2017. Multiplex real-time polymerase chain reaction (PCR) was used to test for respiratory viruses. Multivariate logistic regression was used to compare mortality among patients with HCoV 229E and HCoV OC43 infections. The main outcome was 30-day all-cause mortality. Of 8071 patients tested, 1689 were found to have a respiratory virus infection. Of these patients, 133 had HCoV infection, including 12 mixed infections, 44 HCoV 229E infections, and 77 HCoV OC43 infections. HCoV 229E infections peaked in January and February, while HCoV OC43 infections occurred throughout the year. The 30-day all-cause mortality was 25.0% among patients with HCoV 229E infection, and 9.1% among patients with HCoV OC43 infection (adjusted odds ratio: 3.58, 95% confidence interval: 1.19–10.75). Infections with HCoVs 229E and OC43 appear to have different seasonal patterns, and HCoV 229E might be more virulent than HCoV OC43.

## Introduction

In adults, respiratory virus infections are more frequent than bacterial infections as a cause of community-acquired pneumonia (CAP)^1^. Respiratory viruses are very contagious and are of public health importance^2,3^. The demand for information on the clinical characteristics and prognosis of various respiratory virus infections is likely to increase.

In general, coronavirus infections are asymptomatic in healthy people but they are a common cause of upper respiratory infections (URIs)^4–6^, which may progress to pneumonia^7,8^, Coronaviruses are generically diverse^9^, and may induce distinctive illnesses, including Middle East Respiratory Syndrome (MERS) and Severe Acute Respiratory Syndrome (SARS)^10–13^. Concerns about coronaviruses as an important pathogen with pandemic potential increased with the emergence of SARS-CoV-2, the new coronavirus that is causing the COVID-19 pandemic^14,15^. Coronaviruses became a global issue nowadays.

There are few studies on the epidemiology and prognosis of coronavirus infections among patients with respiratory symptoms seen in emergency departments and hospital inpatients^7,16^. The prognosis of viral infections is influenced by age and comorbidities^17^. A recent study shows the importance of endemic coronavirus in severe respiratory infection in adults^18^ and asymptomatic infection in health care personnel^19^. However, mortality from human coronaviruses (HCoVs) in hospitalized patients has not been well studied, and studies of the prognosis of HCoV infections need to take comorbidities and age into account.

Coronavirus respiratory infections occur primarily in the winter, although the infection can occur at any time of the year^20^. Multiplex PCR can detect multiple respiratory viruses simultaneously^21,22^, providing an opportunity to determine the seasonal patterns of different types of HCoV infection.

We conducted a study to determine and compare the clinical characteristics and mortality associated with HCoV 229E and OC43 infections among patients seen in the emergency department and hospital inpatients.

## Results

**Figure 1:**
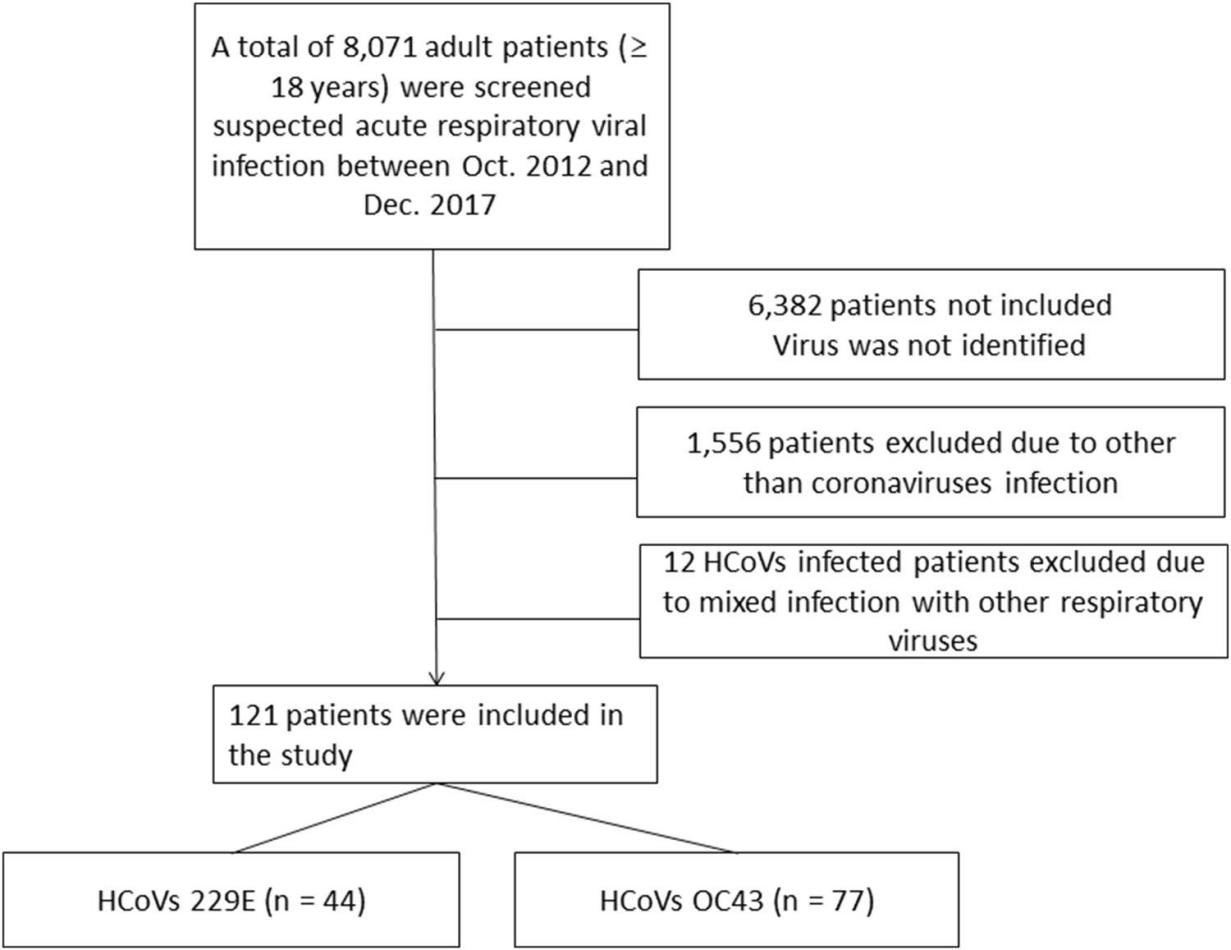
Flow chart showing the selection process of patients with human coronavirus infection.

The process of selecting the patients eligible for inclusion in the analysis is shown in Fig. 1. There were 8071 patients tested for respiratory infections with virus multiplex PCR during the study period, of whom 1689 (20.9%) had identifiable viral infections, 133 (1.6%) had HCoV infection. Among HCoV detected patients, 12 had HCoV infections mixed with other viral infections, and 121 had HCoV mono-infections. Of the 121 patients with HCoV mono-infections, 44 had HCoV 229E infection, and 77 had HCoV OC43 infection. Of the 12 patients with mixed infections one had HCoV OC43 and bocavirus coinfection, one had HCoV 229E and metapneumovirus coinfection, one had HCoV OC43 and parainfluenza type 2 coinfection, one had HCoV OC43 and respiratory syncytial virus A coinfection, one had HCoV 229E and respiratory syncytial virus B coinfection, one had HCoV 229E and rhinovirus A coinfection, four had HCoV 229E and influenza A coinfection, one had HCoV OC43 and influenza B coinfection, and one had HCoV 229E and influenza B coinfection.

Of the 121 patients with HCoV mono-infection, all had chest radiography results, and 72 (59.5%) had chest CT scan results. Thirty-five patients did not have data on arterial blood gas analysis.

### Clinical characteristics

The characteristics of patients with HCoV 229E and HCoV OC43 infections are compared in Table 1. The two groups were similar in terms of age, sex, residence in long-term care facilities and comorbidities, including pneumonia and respiratory bacterial coinfection. Patients with HCoV 229E infection had a higher mean body temperature than patients with HCoV OC43 infection. Although statistics did not reach to significance, a higher proportion of patients with HCoV 229E infection than those with HCoV OC43 infection were admitted to the intensive care unit (22.7% versus 14.2%, P = 0.23), but their mean Pneumonia Severity Index (PSI) scores were similar (101.1 versus 98.7, P = 0.70).

**Table 1:** Characteristics, comorbidities, complications, and outcomes of patients infected with human coronavirus 229E and OC43, October 2012 to December 2017.

Variables	229E (N = 44)	OC43 (N = 77)	P value
Male, n (%)	27 (61.4)	48 (62.3)	0.91
Age (years) mean (SD)	64.3 (16.0)	65.4 (14.1)	0.69
Resident of long-term care facilities, n (%)	2 (4.5)	4 (5.2)	0.87
Malignancy, n (%)	12 (27.3)	19 (24.7)	0.75
Congestive heart failure, n (%)	5 (11.4)	10 (13.0)	0.79
Diabetes, n (%)	15 (34.1)	23 (29.9)	0.63
Liver disease, n (%)	3 (6.8)	6 (7.8)	0.84
Chronic obstructive pulmonary disease, n (%)	3 (6.8)	6 (7.8)	0.84
Asthma, n (%)	3 (6.8)	8 (10.4)	0.51
Body temperature ℃ (SD)	36.7 (0.59)	37.1 (0.68)	< 0.01
Headache	3 (6.8)	0 (0)	0.04
Diarrhea	3 (6.8)	7 (9.1)	0.66
Pneumonia, n (%)	17 (38.6)	25 (32.5)	0.49
Respiratory bacterial infection, n (%)	6 (13.6)	14 (18.2)	0.52
Blood stream infection	7 (15.9)	5 (6.5%)	0.09
Hypoxemia, n (%)	26 (59.0)	44 (57.1)	0.55
PSI	101.1 (31.9)	98.7 (32.2)	0.70
Admission to ICU, n (%)	10 (22.7)	11 (14.2)	0.23
Thirty-day all-cause mortality (%)	11 (25.0)	7 (9.1)	0.03
Sixty-day all-cause mortality (%)	12 (27.3)	11 (14.3)	0.09

*PSI* pneumonia severity index, *ICU* intensive care unit, *SD* standard deviation.

There was no significant difference in bacterial detection between the two groups in sputum or blood cultures (Table 1).

### Seasonal variation

**Figure 2:**
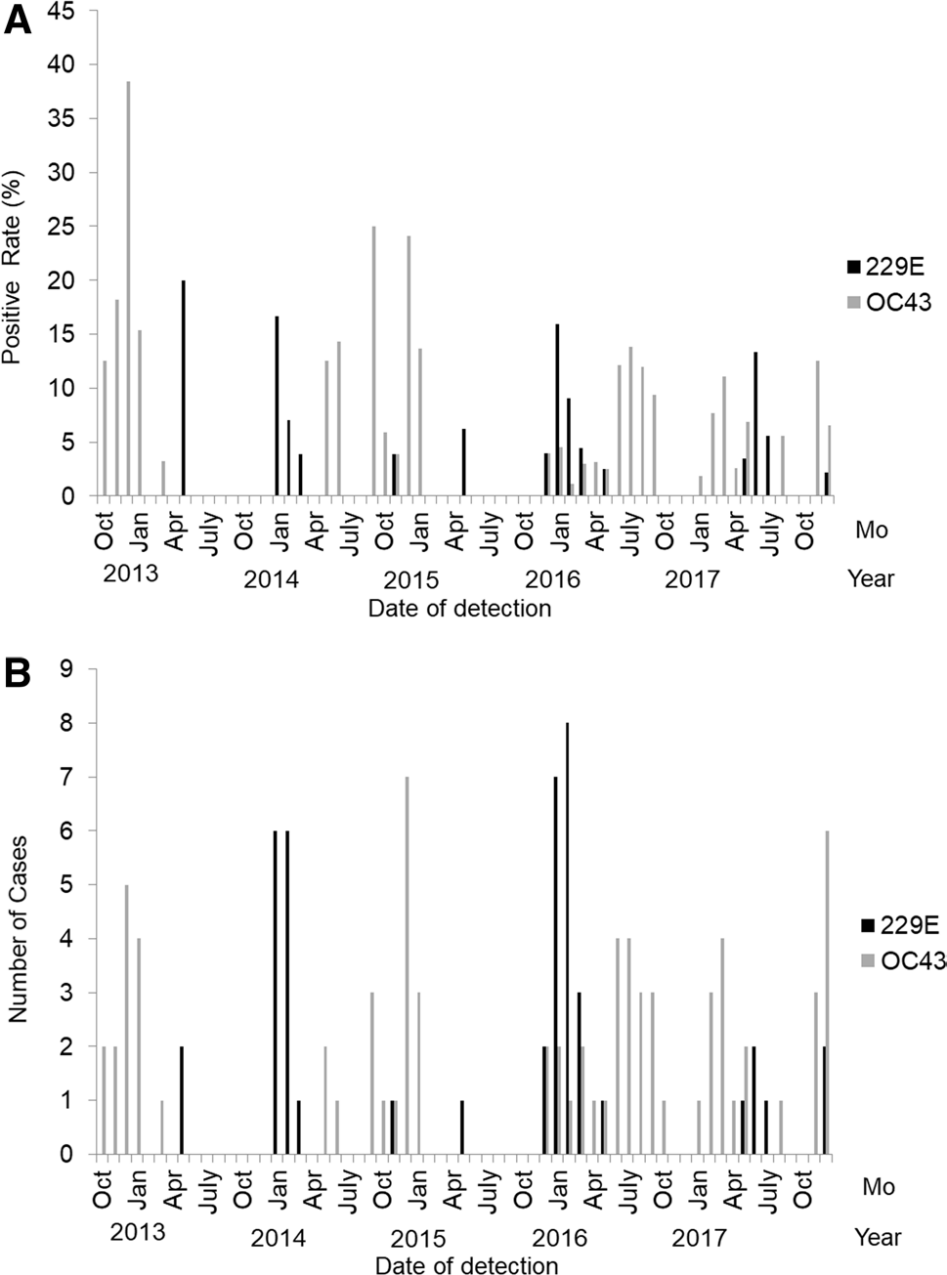
Human coronaviruses (HCoV 229E, black bar and HCoV OC43, gray bar) detected rate (**A**) among virus-infected adult patients and the number of infection (**B**) in each month, October 2012 to December 2017.

There were seasonal peaks in the number of patients admitted with HCoV 229E from January to February in 2014 and 2016, but there were December to January seasonal peaks in admissions of patients with HCoV OC43 infection in 2013 and 2015 (Fig. 2). HCoV OC43 infection could be found throughout the year.

### Mortality

**Figure 3:**
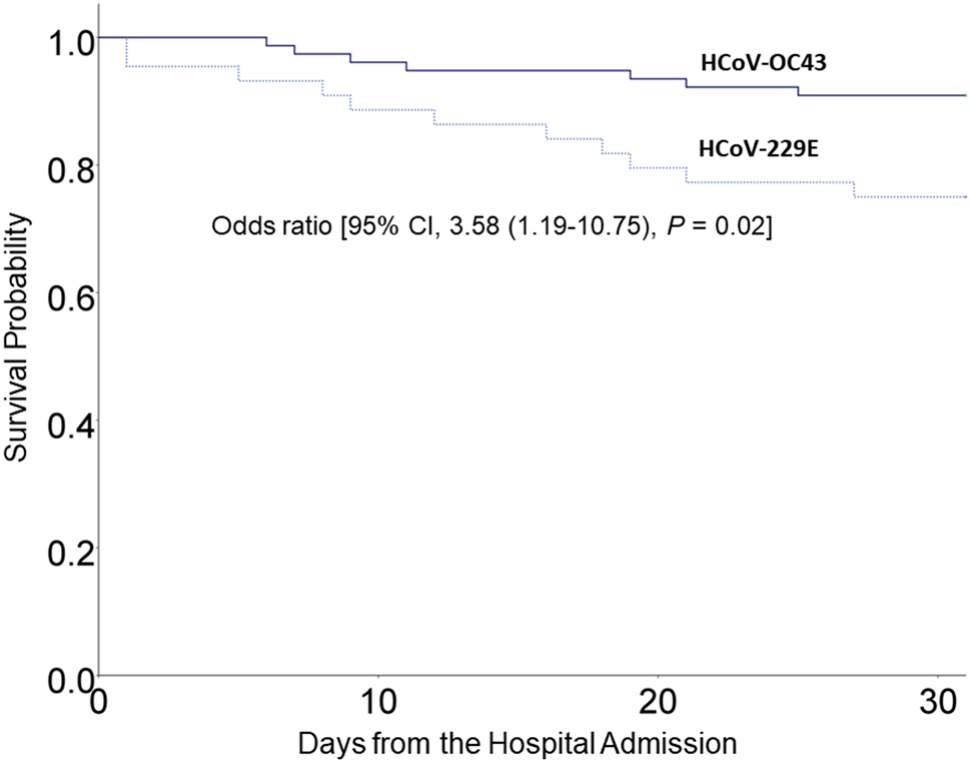
Kaplan–Meier survival curves of 121 adults hospitalized with human coronavirus 229E (dotted line) and OC43 infections (solid line). Patients with HCoV 229E infection had a lower survival rate than those with HCoV OC43 infection. The crude 30-day mortality among patients with human coronavirus 229E and OC43 infections was 25.0% and 9.1%, respectively.

The crude 30-day mortality in patients with HCoV 229E and HCoV OC43 infections was 25.0% and 9.1%, respectively (Fig. 3). The results of the multivariable analysis showed that the risk of death within 30 days (30-day mortality) was higher in patients with HCoV 229E infection than those with HCoV OC43 infection (odds ratio 3.58, 95% confidence interval 1.19–10.75, P = 0.02) (Table 2).

**Table 2:** Factors associated with 30-day all-cause mortality among patients with human coronavirus 229E and OC43 infections.

Variable	Univariate analysis		Multivariate analysis	
	OR (95% CI)	*P* value	OR (95% CI)	*P* value
Age	1.02 (0.98–1.06)	0.17	1.03 (0.99–1.07)	0.14
Sex (men)	1.39 (0.49–3.97)	0.53	1.07 (0.36–3.12)	0.90
Body temperature (°C)	0.92 (0.43–1.98)	0.84	1.28 (0.54–3.02)	0.56
Bacteremia	2.08 (0.57–8.60)	0.30	1.71 (0.38–7.72)	0.48
HCoV 229E	3.33 (1.18–9.34)	0.02	3.58 (1.19–10.75)	0.02

### Bacterial coinfections

Of the 121 HCoV-infected patients, sputum specimens were obtained from 60 (49.6%), and blood-culture specimens from 112 (92.6%) patients. A pathogen in sputum was detected in 20 (33.3%) patients, of which 12 (60.0%) had Gram-positive pathogens, and 8 (40.0%) had Gram-negative pathogens. With regards to blood-culture, 12 (10.7%) patients had positive results, of whom 10 (83.3%) had Gram-positive pathogens, and 2 (16.7%) had Gram-negative pathogens detected on blood culture. *Staphylococcus* *aureus* was the most common pathogen isolated in both sputum and blood specimens (Table 3).

**Table 3:** Bacterial pathogens identified among patients with human coronavirus 229E and OC43 infections according to sample type and virus type.

Pathogen	Sputum		Blood	
	HCoV-229E (n = 44)	HCoV-OC43 (n = 77)	HCoV-229E (n = 44)	HCoV-OC43 (n = 77)
*S. aureus*	3 (6.8%)	5 (6.5%)	2 (4.5%)	3 (3.9%)
*S. pneumoniae*	1 (2.2%)	2 (2.5%)	1 (2.2%)	0 (0.0%)
Other Staphylococcus*	0 (0.0%)	1 (1.3%)	3 (6.8%)	1 (1.3%)
*Klebsiella* species	1 (2.2%)	3 (3.8%)	0 (0.0%)	0 (0.0%)
*Pseudomonas*	1 (2.2%)	1 (1.3%)	0 (0.0%)	0 (0.0%)
other *Enterobacteriaceae*	0 (0.0%)	2 (2.6%)	1 (2.2%)	1 (1.3%)

*Other staphylococci in the blood are regarded as not true pathogens.

## Discussion

Of 8071 patients with respiratory symptoms who were seen in the emergency department or hospitalized and that were tested for respiratory viruses, 1689 (20.9%) had identifiable viral infections. HCoV was found in 133 patients (1.6%). This suggests that HCoVs account for a significant proportion of respiratory viral infections in hospitalized respiratory virus-infected patients. In this study, the prevalence of HCoV infection was slightly lower than that found among patients with CAP in other studies^23,24^. As this study was conducted among patients admitted to the emergency room or hospitalized with respiratory symptoms, the reports of the prevalence of HCoV 229E and HCoV OC43 infection found in this study should be limited to these patients. There are growing evidence of the importance of HCoV infection in healthy individuals and health care personnel^18,19^.

The 30-day mortality rate of patients with HCoV infection was 25.0% for HCoV 229E and 9.1% for HCoV OC43. HCoV 229E infections peaked in January and February of 2014 and 2016.

In one study, the incidence of HCoV was 21.7% among healthy, elderly patients with respiratory symptoms, 21.3% in high-risk patients, and 8.2% in inpatients^7^. A study of patients with chronic obstructive pulmonary disease (COPD) and exacerbated respiratory symptoms found that 14% had HCoV 229E or HCoV OC43 infection^25^. In this study, HCoV 229E and HCoV OC43 were found only in 1.6% of patients with respiratory symptoms. However, in a study done in Edinburgh, 1.25% of the respiratory samples tested using multiplex were positive for HCoV 229E or HCoV OC43^20^, which is lower than that found in our study. The variability in the proportion of individuals with HCoV detected across studies suggests that the prevalence of HCoV infection varies depending on the characteristics of the study participants or the geographic region.

In this study, while HCoV 229E showed clear peaks in January to February 2014 and January to February 2016, but the frequency of HCoV OC43 infections seemed to display December to January seasonal peaks in 2013 and 2015. Like the present study, other studies have shown biennial peaks of HCoV OC43 in winter, and a higher incidence of HCoV 229E infection in winter^20,26,27^. However, HCoV OC43 infection could be found in throughout the year. Therefore, it may be difficult to define the occurrence of HCoV infections to specific seasons.

In this study, among patients with HCoV-infection, headache was only reported by some patients infected with HCoV 229E, whereas the incidence of diarrhea was similarly observed in both 229E and OC43 infections. The prevalence of bacteremia was higher among patients with HCoV 229E infection than those with HCoV OC43 infection, but this difference was not statistically significant. In this study, the prevalence of pneumonia was similar among patients with HCoV 229E infection and those with HCoV OC43 infection, and was higher than that observed in other studies^7^. The mean pneumonia severity index score among hospitalized patients with HCoV OC43 and HCoV 229E infections was 99 and 101, indicating that the patients with HCoV infection had severe disease. In contrast to this study, previous studies have found a higher prevalence of pneumonia among patients with HCoV OC43 infection than in those with HCoV 229E infection^7^. In contrast to previous studies, in our study, admission to the intensive care unit was more frequent among HCoV 229E infections than in those with HCoV OC43 infections^7^.

In our study, we did not find any patients with coinfections with two different types of HCoV. However, 9.0% of the patients with HCoV infections had coinfections with other respiratory viruses. The most observed coinfections were influenza A, which accounted for four of the 12 coinfections. In contrast to our study, other studies have reported a prevalence of HCoV 229E or HCoV OC43 coinfection, ranging from 20 to 30%^27,28^.

Mortality rates of 28–35% have been reported among patients with MERS^29,30^, and 6–15% among patients with SARS and most deaths have occurred in individuals aged older 60 years^31,32^. If the mortality rate could be translated into the virulence of viruses, MERS are more virulent than SARS. In our study, patients with HCoV 229E infection had a higher mortality rate than those with HCoV OC43 infection. It would be important to have more information on the severity of the coronavirus infection with controlling confounders. In this study, HCoV 229E had more than 3 times higher mortality than HCoV OC43 after adjusting confounders. This suggests that HCoV 229E might be more virulent than HCoV OC43.

This study has some limitations. We were unable to test patients for HCoV NL63 or HCoV HKU1 virus because test kits only detected HCoV 229E and HCoV OC43. Therefore, we did not have information on HCoV NL63 and HCoV HKU1 infection which would provide a better picture of HCoV infection. Furthermore, due to other respiratory viruses not included in the PCR used, virus co-infection could not be completely ruled out. We have performed a multivariate analysis that controls variables, but a small sample size may not fully adjust for important confounders affecting prognosis. The study did not include patients with mild respiratory symptoms or patients who were asymptomatic, thus patients with few comorbidities and a good prognosis are likely to have been excluded, preventing the study results from providing a comprehensive clinical picture of HCoV infection. In addition, the prevalence of HCoV infections among the patients tested for respiratory viruses may have been underestimated because lower airway samples were not tested. Data were collected retrospectively, and missing data and inadequate documentation may have resulted in biases in the study results.

## Conclusion

Among hospitalized patients with suspected respiratory infection, 1.6% had HCoV infection. HCoV 229E infections and HCoV OC43 infections appear to have seasonally different patterns. HCoV 229E infection might cause more severe disease than HCoV OC43 infection in adults.

## Materials and methods

### Study population and setting

We conducted a retrospective cohort study of adults hospitalized with HCoV infections. All patients aged ≥ 18 years admitted with suspected respiratory virus infections between October 2012 and December 2017 were considered. Virus multiplex PCR was performed if physicians suspected respiratory virus infection. Patients with no viral infections identified, viral infections other than HCoV infections, and mixed viral infections were excluded.

The study was approved by the institutional review board (IRB) at Dongsan Hospital, Keimyung University School of Medicine (2019-08-026). The IRB waived the requirement for informed consent, and the study was conducted in compliance with the Declaration of Helsinki.

### Definitions

An upper respiratory infection was defined as the presence of one or more of the following respiratory symptoms: cough, sputum production, rhinorrhea, sore throat, or dyspnea. Pneumonia was defined as the presence of a new or progressive infiltrate found using either chest radiography or chest CT scan, in addition to two or more of the following: fever, sputum production, rhinorrhea, sore throat, dyspnea, or a diagnosis of pneumonia by the attending physician. The outcome was all-cause mortality up to 30 days after hospital admission. The HCoVs detection rate was calculated using all patients who visited the hospital with respiratory symptoms and performed respiratory virus PCR as the denominator, and the HCoVs detection cases as the numerator. The HCoV co-detection percentage was obtained using total HCoV detection cases as the denominator, and respiratory viruses detection cases other than HCoVs as the numerator.

### Specimens

Nasopharyngeal specimens were obtained using flocked swabs and stored and transported using the universal transport medium, as described elsewhere^33^. We routinely ordered sputum and blood culture. However, sputum and blood-culture specimens were obtained in 60 and 112 cases, respectively, from 121 HCoV-infected patients.

### Respiratory virus testing

Respiratory virus (RV) testing was performed to detect the following viruses: adenovirus (A–F), influenza viruses A and B, respiratory syncytial virus (RSV) A and B, parainfluenza viruses 1 to 3, Rhinovirus, metapneumovirus, coronavirus 229E, coronavirus OC43, and bocavirus. During the RV test, an internal control was added to each specimen to check the entire process from nucleic acid extraction to PCR. A Real-Q RV Detection Kit (BioSewoom, Seoul, Korea) was used, according to the manufacturer's instructions described in elsewhere^34^. The Real-Q multiplex PCR kit was sensitive to detect respiratory viruses in respiratory samples, and the kit also performed well in detecting virus co-infection^34^.

### Microbiology

Microorganisms in samples obtained from sputum, or blood within 24 h of admission were investigated. Sputum samples were cultured in a semi-quantitative manner, and pathogens were identified when a predominant microorganism was detected from group 4 or 5 sputa, according to Geckler’s grading system^35^. All isolates were determined using automated bacterial identification (VITEK2; bioMerieux Inc., Lyon, France).

### Data collection

This study was performed at Keimyung University Dongsan Hospital, a 867-bed, tertiary care teaching hospital in Daegu, Republic of Korea. If a patient had an episode of acute respiratory infection at an emergency department or outpatient clinic or within 2 days during admission, he or she underwent multiplex RT-PCR (Reverse transcription polymerase chain reaction) testing. Adult patients (≥ 18 years of age) who had a multiplex RT-PCR test between October 2012 and December 2017 were identified by searching electronic medical records. We collected clinical data from the electronic medical records on general patient characteristics, co-morbidities, presenting symptoms, lower respiratory tract complications, provision of supplemental oxygen therapy and/or ventilatory support, length of hospital stay, and all-cause mortality. We also collected data on all patients’ chest radiography, including the radiologists’ reports, chest computerized tomography scans (if available), and routine blood test results. Sputum samples were collected for bacterial culture preparation at admission and during hospitalization. Blood cultures were also performed when indicated. Pneumonia severity index (PSI) score was collected every admitted patient. We contacted patients or their families by phone to identify survival and clinical information if the patients were not followed up regularly.

### Statistical analysis

Baseline characteristics (including age, sex, residency in a long-term care facility, comorbidities, presenting symptoms, and complications) at the date of admission were compared between 229E and OC43 infection using descriptive statistics, such as proportion and means (standard deviation, SD). A Chi-squared test was used for comparison between categorical variables, and independent *t* tests, for comparison between continuous variables. Headache was compared using Fisher’s exact test. The univariate and multivariate logistic regression models were used to evaluate the risk of death from HCoVs infection between their types. Adjustments were made for sex, age, body temperature and bacterial co-detection. The selection of covariates for the model building was based on clinical relevance and availability. Variables showing P value less than 0.2 in univariate analysis also included in the models except for age and sex. *P* values < 0.05 were considered statistically significant. All statistical analyses were performed using IBM SPSS V.21.0.
